# Radiation therapy advanced practice – commentary

**DOI:** 10.1002/jmrs.29

**Published:** 2013-11-20

**Authors:** Jenny Cox

**Affiliations:** Royal North Shore Hospital – Northern Sydney Cancer Centre Pacific Highway, St Leonards, NSW 2065, Australia; University of Sydney – Faculty of Health Sciences PO Box 170, Lidcombe, NSW 1825, Australia

Re: Monk CM, Wrightson SJ, Smith TN. An exploration of the feasibility of radiation therapist participation in treatment reviews. *J Med Radiat Sci* 2013; **60**(3): 100–7.

It is pleasing to see another piece of research aimed at informing the radiation therapy (RT) advanced practice (AP) debate.^1^ The formal introduction of AP in radiography (diagnostic and therapeutic) occurred at least as early as 2000 in the United Kingdom, instigated by the National Health System (NHS) because of a national shortage of medical staff.^2^,^3^ Diagnostic radiography AP was predominantly in the area of image review carried out by experienced and often trained radiographers, ranging from Red Dot processes to formal review of images.^4^–^6^ RT AP was more varied and was tailored to suit the needs of individual departments of radiation oncology. These needs are usually related to a shortage of radiation oncologists (RO) in specific specialty areas, where experienced RTs could step in and help to move the patient more smoothly through the system. Postgraduate education in collaboration with the clinical place was then introduced to formalize the advanced roles.

While RT AP in the United Kingdom (UK) was encouraged by the NHS, in Australia and New Zealand it is a ‘bottom-up’ approach, being led by RTs and supported only in certain supportive departments of radiation oncology. This means that while individual RTs might struggle to convince their supervisors about their advanced attributes, some formalized research projects have been carried out, aimed at assessing RTs’ ability to advance their practice and providing validation of the new or proposed roles. This provides an evidence-based approach to the introduction of RT AP roles.

The recent article by Monk, Wrightson and Smith^1^ presents additional evidence to inform the introduction of RT AP in the area of patient treatment review. The research project contained two arms: an audit of patients on treatment to determine the frequency of side effects detected by the RO and the interventions carried out, and a survey of the local ROs to determine their support (or not) of RT reviewers. Similar to Shi et al.^7^ in Singapore, Monk et al. found that radiation oncologist reviews led to medical interventions for fewer than 35% of patients receiving radiotherapy to the prostate, breast, bladder and skin regions. The fact that prostate and breast treatments, with low levels of medical intervention, are a large proportion of the Australian radiotherapy workload provides evidence to support the AP role of RT patient reviewer in these areas. Additional evidence was provided by Acharya et al.^8^ who found no significant difference in the ranking of skin toxicities in the breast area between RTs and ROs. UK researchers also found in a large sample study that patients being treated for breast cancer were very satisfied with their RT reviewers.^3^ There is currently a trial under way at the Northern Sydney and Illawarra Cancer Centres, New South Wales, where patients' recorded side effects and interventions at weekly RT breast cancer patient reviews are being compared with RO review results.^9^

An interesting difference between the findings of Monk et al.^1^ and the Singapore work^7^ is the opinions of the ROs involved. Those in Singapore were willing to cede some review duties to RTs, whereas those in Newcastle were not. This difference warrants further investigation using qualitative methodology to explain the reasons for the lack of support from some ROs. There is no doubt that ROs take responsibility for the overall management of a patient's treatment, but qualified RTs already perform many independent roles when imaging, planning and treating patients. In the days of film-based portal imaging, a check film was taken, developed and reviewed by the RO before the next treatment fraction, at best, and later in some cases, with field movement then made if necessary. The introduction of new technology such as image-guided RT, which allows close to real-time correction of patient isocentre locations, has removed the checking role of on-treatment images from the RO. Isocentre checking was passed to the RTs, who initially felt the need for extra training but now incorporate it into their routine daily duties with no RO supervision.^10^,^11^ Comparative studies have shown that there is no significant difference between the performance of RTs and ROs in this role.^12^–^14^ There was no resistance from the ROs to this transfer of roles, presumably because the advantages of immediate correction of field placement errors were irrefutable. With the growing ‘mismatch’ between RO and patient numbers, patient review will be another area where responsibilities will need to be transferred, and the research literature strongly suggests that RTs have the necessary attributes to take on this role.

There could also be industrial benefits from the creation of a RT reviewer position. RT has always had a comparatively flat career structure, with most promotional positions involving administrative rather than clinical roles. Technical advances have led to some specialist roles in the treatment planning and imaging areas. The creation of positions such as patient reviewer RTs would reward those RTs who have a strong patient care focus and acknowledge the importance of a strong RT/patient relationship.

I believe that no change in oncology roles has been supported by such a large body of research evidence as the RT treatment reviewer role, so it is disappointing that it has not been formally and widely implemented in Australia.
